# A nationwide comprehensive genomic profiling and molecular tumor board platform for patients with advanced cancer

**DOI:** 10.1038/s41698-025-00858-0

**Published:** 2025-03-10

**Authors:** Pieter-Jan Volders, Philippe Aftimos, Franceska Dedeurwaerdere, Geert Martens, Jean-Luc Canon, Gabriela Beniuga, Guy Froyen, Jacques Van Huysse, Rebecca De Pauw, Hans Prenen, Suzan Lambin, Lore Decoster, Freya Vaeyens, Sylvie Rottey, Pieter-Jan Van Dam, Lynn Decoster, Annemie Rutten, Max Schreuer, Siebe Loontiens, Joni Van der Meulen, Jeroen Mebis, Kristof Cuppens, Sabine Tejpar, Isabelle Vanden Bempt, Jacques De Grève, David Schröder, Cédric van Marcke, Marc Van Den Bulcke, Evandro de Azambuja, Kevin Punie, Brigitte Maes

**Affiliations:** 1 Laboratory for Molecular Diagnostics, Jessa Hospital, Hasselt, Belgium; 2 Faculty of Medicine and Life Sciences, LCRC, University of Hasselt, Hasselt, Belgium; 3 Department of Biomolecular Medicine, Ghent University, Gent, Belgium; 4 Clinical Trials Conduct Unit, Institut Jules Bordet—Université Libre de Bruxelles, Hôpital Universitaire de Bruxelles, Brussels, Belgium; 5 Department of Pathology, AZ Delta Hospital, Roeselare, Belgium; 6 Department of Laboratory Medicine, AZ Delta Hospital, Roeselare, Belgium; 7 Department of Medical Oncology, Grand Hôpital de Charleroi, Charleroi, Belgium; 8 Institut de Pathologie et de Génétique, Charleroi, Belgium; 9 Department of Pathology, AZ Sint-Jan Brugge, Bruges, Belgium; 10 Department of Pulmonology, AZ Sint-Jan Brugge, Bruges, Belgium; 11 Department of Oncology, Antwerp University Hospital, Edegem, Belgium; 12 Center for Oncological research, University of Antwerp, Wilrijk, Belgium; 13 Department of Pathology, University Hospital Antwerp, Edegem, Belgium; 14 Department of Medical Oncology, University Hospital Brussels, Brussels, Belgium; 15 Laboratory for Medical and Molecular Oncology, Vrije Universiteit, Brussels, Belgium; 16 Centre for Medical Genetics, University Hospital Brussels, Brussels, Belgium; 17 Department of Medical Oncology, Ghent University, Ghent, Belgium; 18 CellCarta, Antwerp, Belgium; 19 Department of Pulmonology, AZ Turnhout, Turnhout, Belgium; 20 Department of Medical Oncology, ZAS hospitals, Antwerp, Belgium; 21 Department of Medical Oncology, ASZ Aalst, Aalst, Belgium; 22 Molecular Diagnostics, Ghent University Hospital, Ghent, Belgium; 23 Cancer Research Institute Ghent, Ghent University, Ghent, Belgium; 24 Department of Medical Oncology, Jessa Hospital, Hasselt, Belgium; 25 Department of Pulmonology and Thoracic Oncology, Jessa Hospital, Hasselt, Belgium; 26 Digestive Oncology, University Hospitals KU Leuven, Leuven, Belgium; 27 Department of Human Genetics, University Hospitals KU Leuven, Leuven, Belgium; 28 Institute for Experimental and Clinical Research, UCLouvain, Brussels, Belgium; 29 Department of Medical Oncology, Institut Roi Albert II, Cliniques universitaires Saint-Luc, Brussels, Belgium; 30 Sciensano, Brussels, Belgium; 31 Institut Jules Bordet, Hôpital Universitaire de Bruxelles and l’Université Libre de Bruxelles, Brussels, Belgium

**Keywords:** Predictive markers, Molecular medicine, Tumour biomarkers, Targeted therapies, Cancer genomics

## Abstract

The Belgian Approach for Local Laboratory Extensive Tumor Testing (BALLETT) study assessed the feasibility of using comprehensive genomic profiling (CGP) in clinical decision-making for patients with advanced cancers. This multi-center study enrolled 872 patients from 12 Belgian hospitals. CGP was performed on tumor tissues using a standardized CGP panel (523 genes) across nine laboratories with success in 93% of patients and a median turnaround time of 29 days. Actionable genomic markers were identified in 81% of patients, substantially higher than the 21% using nationally reimbursed, small panels. A national molecular tumor board (nMTB) recommended treatments for 69% of patients, with 23% receiving matched therapies. Reasons for non-compliance were highly variable across clinical sites. Overall, BALLETT demonstrates the feasibility of implementing decentralized CGP and its potential to identify actionable targets in most patients with advanced cancers. BALLETT reinforces CGP’s utility and emphasizes the importance of collaboration, standardization, and addressing implementation challenges.

## Introduction

The complexity and heterogeneity of advanced-stage cancer in the context of increasing availability of biomarker-specific and tumor-agnostic systemic therapy provide significant challenges in treatment decision-making for patients with advanced disease. Over the past decade, the advent of comprehensive genomic profiling (CGP) through next-generation sequencing (NGS) has emerged as a promising avenue to unravel the intricate genomic landscape of tumors, offering unprecedented insights into the continuously broadening spectrum of potential therapeutic targets^1^. Next, to single-nucleotide variants (SNVs), short insertions and deletions (indels), copy-number variants (CNVs), and gene fusions, CGP also refers to the assessment of genomic signatures with increasing relevance in predicting response to targeted drugs and immunotherapy drugs such as microsatellite instability (MSI), tumor mutational burden (TMB), and homologous recombination deficiency (HRD)^2–5^. The incorporation of genomic data into clinical decision-making has consistently proven effective in customizing treatment approaches for individual patients, marking the advent of precision oncology^6–15^. A recent, comprehensive review demonstrated that progression-free survival (PFS) and overall survival (OS) were significantly longer among patients who were matched to targeted treatment across tumor types, confirming that genome profiling-based treatment can have an impact on survival across tumor types^16^.

Despite positive evidence for the clinical efficacy of CGP in numerous studies, widespread adoption is hindered by budgetary constraints and other barriers^17^. In a recent European Society for Medical Oncology (ESMO) study, Bayle et al. highlighted the low access to CGP across Europe^18^. Small NGS panels are more widely used due to their lower cost and complexity, but the scope for identifying actionable targets is also smaller. While techniques like whole exome sequencing, whole genome sequencing, and whole transcriptome sequencing offer the most comprehensive insights, their implementation often faces even greater challenges in terms of cost, infrastructure, and data interpretation.

To address these challenges and to provide a framework for CGP at the national level, we initiated the Belgian Approach for Local Laboratory Extensive Tumor Testing (BALLETT) study, a large-scale study with pragmatic recruitment based on minimal eligibility criteria, aiming to enhance treatment options and outcomes for patients with advanced cancers. BALLETT is part of a nationwide initiative by the PRECISION working group of the Belgian Society of Medical Oncologists (BSMO)^19^. The BSMO PRECISION initiative seeks to bridge the financial gap by partnering with industrial stakeholders and the Belgian public healthcare scientific institution Sciensano to make CGP more accessible, ultimately translating genomic insights into clinical benefits for Belgian patients with metastasized cancer. The BSMO genomic profiling studies, including BALLETT and GeNeo^20^, aim to improve and democratize access to CGP and provide evidence-based treatment recommendations to improve access to biomarker-specific systemic treatment for patients with advanced solid tumors.

Crucially, the BSMO genomic profiling studies employed a multifaceted approach, combining the genomic information of comprehensive profiling with the collective expertise of a national molecular tumor board (nMTB). Patients enrolled in the study received consolidated recommendations by the nMTB based on their CGP results, a practice consistently demonstrating clinical efficacy^21,22^. The collaborative effort of expert oncologists, pathologists, geneticists, molecular biologists, and bioinformaticians within the nMTB serves as a vital link between genomic findings and actionable clinical decisions.

A unique feature of the BALLETT study, distinguishing it from the many similar precision oncology projects, lies in its objective to implement the CGP broadly and uniformly across a consortium of nine Belgian local NGS laboratories, using a fully standardized methodology. In contrast to study designs with central CGP testing, this approach seeks to make the expertise derived from CGP available in local oncology centers, situated close to the clinicians and the patients. In addition, this approach amplifies the potential for broader access to CGP, thereby increasing the chances of reaching a wider patient population.

In this manuscript, we present the BALLETT study design, methodology, and results. We discuss the detected variants and biomarkers, their actionability, and the uptake of the CGP-based treatment recommendations. Furthermore, we provide insight into the real-world challenges and limitations associated with integrating CGP into clinical practice, thereby offering guidance for other institutions seeking to adopt similar standardized approaches to make precision medicine an accessible reality for patients worldwide.

## Results

### Patient recruitment and CGP success rate

**Fig. 1:**
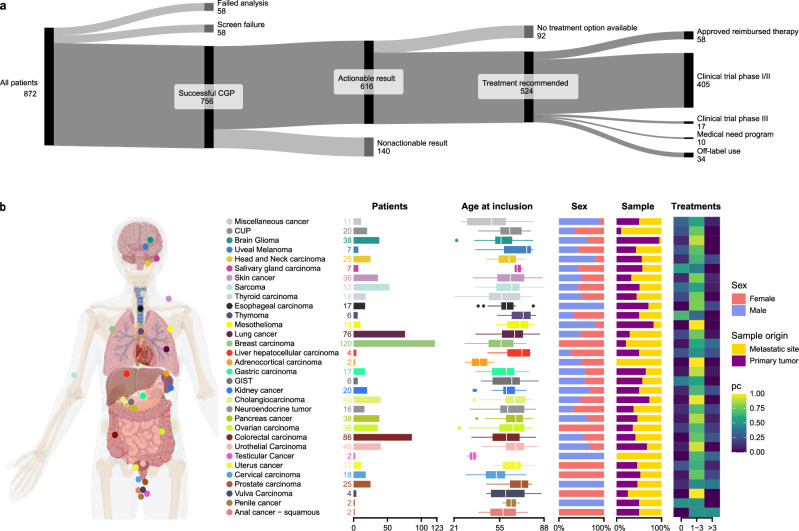
Patient characteristics and study flow. **a** General overview of the BALLETT study recruitment, success rate, and recommendations. **b** Overview of the participants’ characteristics and tumor types.

Eight hundred seventy-two patients consented to this study between May 2021 and October 2023 (Table 1). For 58 patients (7%), no CGP analysis was performed due to insufficient tissue, DNA, or RNA, resulting in screen failure (Fig. 1a). A portion of these patients might have benefited from liquid biopsy CGP testing; however, this approach was not included in the study design. For the 814 remaining patients, CGP was carried out. Repeat analysis was performed for 33 patients. Results were interpretable for 756 patients, giving an overall CGP success rate of 93% (Fig. 1b). The age of the sample was not a significant indicator of success (*p* = 0.0645, logistic regression). A broad range of 32 different tumor types was included with the most frequent being breast cancer (*n* = 123), colorectal cancer (*n* = 87), lung cancer (*n* = 77), sarcoma (*n* = 56), cholangiocarcinoma (*n* = 42), urothelial cancer (*n* = 41), pancreatic cancer (*n* = 41), glioma (*n* = 39), skin cancer (*n* = 39), ovarian cancer (*n* = 37), prostate cancer (*n* = 27), and head and neck cancer (*n* = 26). Histological subtypes are listed in the supplementary information (Supplementary Fig. S1).

**Table 1:** Characteristics of all patients with successful CGP

Age at inclusion, years	
Median	62
Range	21–88
Sex	
Male	330 (44%)
Female	426 (56%)
ECOG performance status	
0	136 (18%)
1	343 (45%)
2	32 (4%)
Missing	245 (32%)
Metastasis at inclusion	
No (locally advanced)	94 (12%)
Yes	662 (88%)
Missing	0 (0%)
Number of metastatic sites	
Median	2
Range	1–20
Missing	96
Number of previous therapy lines for advanced disease	
Median	1
Range	0–11
Missing	0

### CGP was successful across all lab consortium partners

**Fig. 2:**
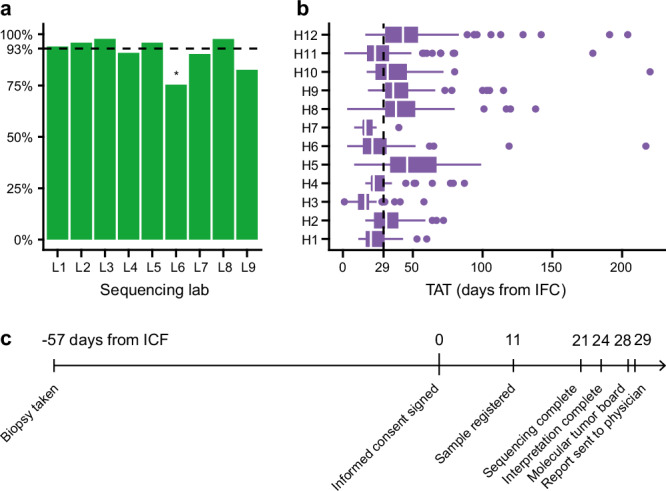
CGP success rate, turnaround times, and timeline. **a** CGP success rate across the different participating labs. The overall success rate was high (93%). **b** Turnaround time (TAT) at the participating sites. TAT is measured as the days between signing the informed consent form and delivering the report to the treating clinician. The median TAT was 29 days, yet differences between the sites are apparent. **c** Timeline of the patient trajectory indicating key milestones with median values in days as assessed for all patients.

CGP success rates in the different tumor types ranged from 72% to 100% (Supplementary Fig. S2). The lowest success rates are observed for uveal melanoma and gastric cancer (72% and 74%, respectively). This might be explained by the generally smaller biopsies available for CGP in these tumor types. The success rate of CGP was not significantly different across the nine NGS labs, except for NGS lab #6 (*p* = 0.0015 by logistic regression). Despite the standardization efforts within the consortium, the CGP success rate was significantly lower (76%) in this lab. This observation may be attributed to local variability factors (e.g., DNA extraction method, tissue preparation procedures, inter-operator variability, etc.) (Fig. 2a).

The median turnaround time (TAT) from inclusion in the study (i.e., informed consent signed) to the nMTB report was 29 days (Fig. 2b, c), and 95% of the reports were available within 66 days of the informed consent. The median TAT differed significantly between hospitals in the study (range 18–45 days, *p* < 0.0001 by ANOVA).

### Molecular alterations and genome-wide biomarkers

**Fig. 3:**
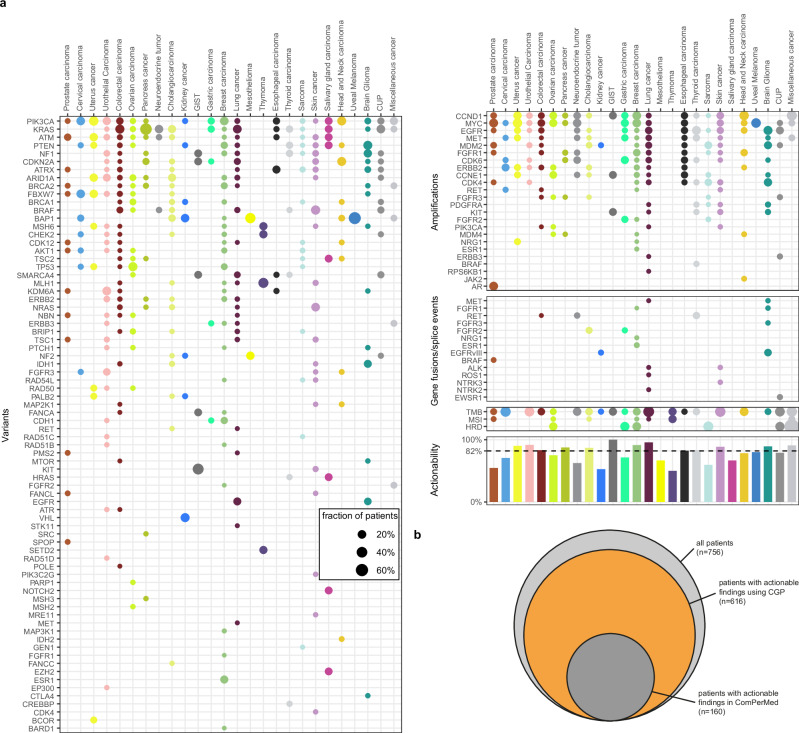
Overview of actionable findings. **a** Frequency of actionable genomic alterations and biomarkers across different tumor types in the BALLETT study. **b** Comparison of actionability using CGP versus standard-of-care gene panels in Belgium.

In the 756 obtained CGP profiles, 1957 pathogenic or likely pathogenic SNVs or indels, 80 pathogenic gene fusions, and 182 amplifications were identified across 276 different genes. The ten most frequently altered genes were *TP53* (370 alterations in 46% of patients), *KRAS* (*n* = 95, 13%), *APC* (*n* = 95, 9%), *PIK3CA* (*n* = 92, 11%), *TERT* (*n* = 57, 8%), *EGFR* (*n* = 41, 4%), *RB1* (*n* = 40, 5%), *ARID1A* (*n* = 39, 5%), *PTEN* (*n* = 39, 5%), and *NF1* (*n* = 34, 4%) (Supplementary Fig. S3). Alteration frequencies per tumor type are illustrated in Supplementary Fig. S4. The median number of alterations per patient was three (range, 0–30, Supplementary Fig. S5). In addition, 124 patients (16%) had a TMB-high score. A high TMB was most frequently observed in lung cancer, melanoma, miscellaneous cancer, CUP, cervical carcinoma, and urothelial carcinomas (Fig. 3). For eight patients, an MSI-high status was detected; all eight also exhibited a high TMB.

HRD status was also analyzed for 100 patients included after April 2023; 11 (11%) showed a positive result, including five breast and two ovarian carcinomas (Fig. 3a). Amongst these HRD-positive cases, two cases had (likely) pathogenic variants in *BRCA1/BRCA2* and 3 had (likely) pathogenic variants in other homologous recombination repair (HRR) genes (*ATM*, *BARD1* and *BRIP1* + *PARP1*).

### Actionability

In the 756 obtained CGP profiles, 1086 actionable markers were identified. Six hundred sixteen patients displayed at least one actionable result, accounting for an overall 81% actionability. Overall actionability varied according to the tumor type (Fig. 3a). Actionable markers comprised 779 SNVs/indels, 129 amplifications, 35 gene fusions, all previously described genome-wide markers predictive for immune therapy (TMB, MSI, *n* = 124), and HRD (*n* = 11). Their distribution across tiers with strong clinical significance (IA, IB) and potential clinical significance (IIC and IID) differed between tumor types (Supplementary Fig. S6). For 311 patients (41%), more than one actionable alteration was found. For 104 patients (14%), both an actionable alteration and an immunotherapy biomarker were demonstrated.

Actionable markers were found in a total of 88 different genes. To evaluate the additional benefit of CGP testing, we estimated the number of patients for whom actionable results would have been obtained using the standard-of-care diagnostic gene panels in Belgium by taking only those genes into account that are included in the local guidelines (ComPerMed). With standard-of-care panels, actionable results would have been obtained for 160 patients, accounting for a substantially lower actionability of 21%, compared to the 81% overall actionability considering the full CGP analysis (Fig. 3b).

### nMTB treatment recommendations

Of the 756 patients with successful CGP analysis, 524 (69%) received at least one treatment recommendation by the nMTB based on their CGP profiles (Supplementary Fig. S7). The full list of biomarker—drug class matches that resulted in a treatment recommendation is provided in Supplementary Table S4. This table also includes the type of highest-priority treatment recommendation and the actionability according to ComPerMed versus CGP at the patient level.

Most often, this concerned a recommendation to participate in a biomarker-driven clinical trial (*n* = 422). The remaining treatment recommendations were approved treatments (*n* = 58), off-label drug use (*n* = 34), or participation in a medical need program (*n* = 10) (Fig. 1b). For 92 patients (15%), actionable CGP results were identified, but no treatment recommendation could be made by the nMTB, mainly due to a lack of drug approval in Belgium (e.g., for PI3K inhibitors) or clinical trials in Belgium or Europe. An overview of the actionable variants that did not lead to a treatment recommendation is provided in Supplementary Table S5.

### Uptake of treatment recommendations

Treatment recommendation uptake information was collected from the participating hospitals for 454 patients. For 104 of 454 patients (23%), the nMTB treatment recommendation was implemented. The main reasons recommendations were not followed included the choice of the treating physician (27%), unavailability of a trial within an acceptable distance (21%), and rapid clinical deterioration of the patient (12%).

**Fig. 4:**
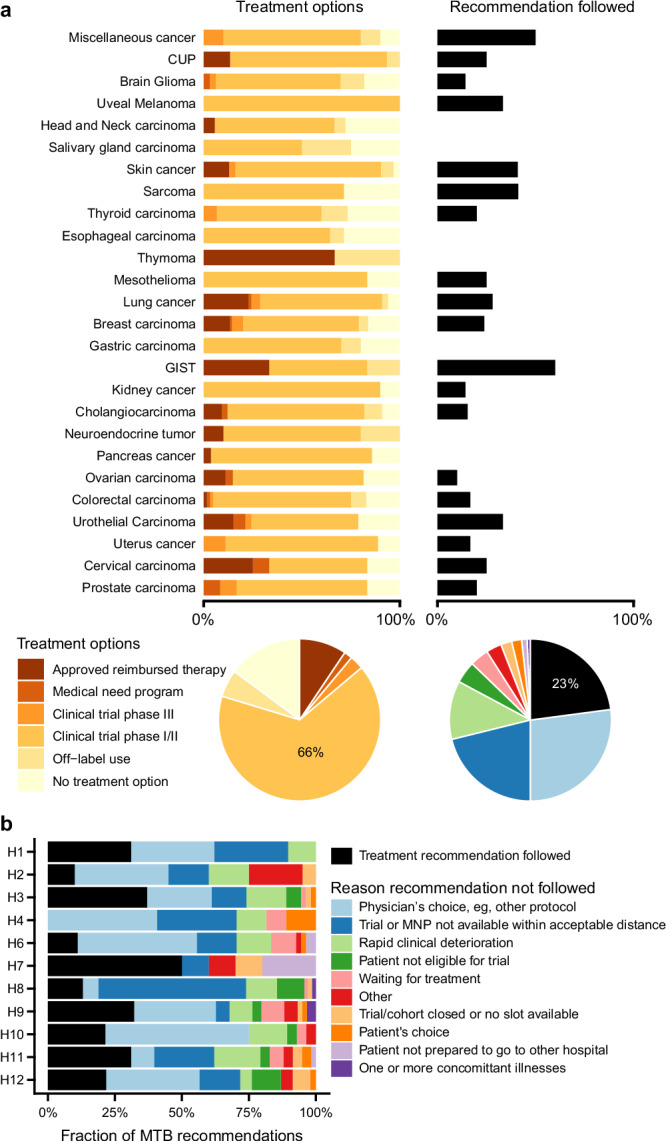
Treatment recommendations and uptake. **a** Relative distribution of treatment recommendation types across tumor types. **b** Uptake rate of the nMTB recommendations and reasons for deviation from recommendations across participating hospitals in the BALLETT study. MNP medical need program.

Both the rate of recommendation uptake and the reasons recommendations were not followed differed significantly between the participating hospitals (*p* < 0.0001 by chi-squared test, Fig. 4, Supplementary Fig. S8). The highest uptake was observed for hospital #7, where only patients with lung cancer were included. Conversely, at hospital #, 40% of the nMTB recommendations were followed. Reasons for the variability of uptake across hospitals are not known.

### Incidental findings of likely germline variants

In 90 patients (12%), a variant in a cancer susceptibility gene (CSG) was identified, as evaluated by molecular geneticists at the local laboratories and the nMTB. The detection of a CSG variant, along with the assessment of its potential germline origin, was documented in the BALLETT nMTB report and communicated to the treating physician to initiate appropriate germline follow-up and clinical genetic counseling. Reanalysis of the data according to the recently updated ESMO recommendations for germline-focused analysis of tumor-only sequencing^23^ revealed 123 CSG variants in 113 patients (15%), fulfilling the ESMO criteria considering all 40 ESMO CSG genes and all tumor types (Supplementary Fig. S9).

## Discussion

While the clinical benefit of CGP has been substantiated in various publications^6–15^, unlocking its full potential to maximize benefit in patients with cancer requires broad and equitable access to diagnostic technologies and innovative drugs^17^. Presently, obstacles to accessing genomic profiling include the substantial costs associated with comprehensive genomic testing—often not covered by existing reimbursement systems—the absence of a feasible pathway for generating evidence to support reimbursement decisions by national healthcare systems, and the complexity of genomic data that necessitates collaborative, multidisciplinary efforts and education of healthcare workers. By setting up BALLETT in a multi-stakeholder approach involving industry, the national healthcare institutions (represented by Sciensano), and a large group of multidisciplinary healthcare workers across Belgium, we aimed to overcome these challenges. We also sought to enhance access by securing financial support from industrial partners, thereby paving the way for the introduction of CGP in real-world clinical oncology. During the ~2.5-year study period, this approach allowed to provide CGP analyses for 814 patients with advanced cancer, recruited in a large number of Belgian oncology centers (*n* = 12). Through this study, patients received CGP-based treatment recommendations from the dedicated nMTB, which was set up by the PRECISION group of BSMO, with the support of Sciensano.

Actionable genomic markers and CGP-based treatment recommendations were provided for 81% and 69% of patients, respectively. These results are in line with other precision oncology projects^6–15^. Furthermore, using standard-of-care diagnostic gene panels in Belgium (ComPerMed), actionable results would have been found in only 21% of patients, compared to 81% with full CGP analysis, demonstrating CGP’s superior potential for tailored treatment recommendations.

We also describe the large collaborative effort of nine NGS laboratories involved in the project. We demonstrate the feasibility of implementing a fully standardized CGP approach, including variant classification and clinical interpretation, across these nine laboratories, thereby ensuring broad accessibility for Belgian patients. The uniform CGP success rates observed across the nine NGS labs, with one exception possibly attributed to local variability factors, underscore the importance and feasibility of implementing robust quality control systems. Addressing factors such as inter-operator differences and variability in sample preparation and DNA/RNA extraction processes is essential to guarantee the accuracy and reliability of CGP results throughout the entire workflow.

The intensive collaboration of all involved NGS labs to collectively deliver high-quality and consistent CGP results undoubtedly enhanced the knowledge and expertise of all laboratory personnel involved, effectively addressing challenges posed by the complexity of genomic data and technology. Similarly, discussing all patients with their CGP results in the nMTB was found to be crucial, not only to allow standardization and prioritization of appropriate treatment recommendations in line with similar projects^6–15^, but also to serve as a valuable framework for education, exchange of expertise, and increasing awareness of oncologists and other healthcare workers. The project, therefore, has contributed to strengthening the confidence of both laboratory personnel and oncologists during local multidisciplinary patient discussions, benefiting patients across the Belgian territory.

Findings of potential germline genetic alterations in 15% of cases highlight an additional layer of benefit from CGP. The nMTB’s recommendation for referral to germline genetic testing and counseling underscores the potential for early identification of patients and families at risk, enabling the timely implementation of cancer preventive measures. Follow-up studies are crucial to evaluate the long-term impact on patients, their families, and the healthcare budget, aligning with the broader discussions in the literature on the ethical implications and outcomes of germline testing in oncology^24,25^.

While the BALLETT study has played a pivotal role in overcoming obstacles associated with precision oncology in Belgium, as was anticipated by BSMO, it is crucial to recognize and address several limitations and persistent challenges. Effectively overcoming these challenges is essential for the sustainable introduction of CGP in healthcare systems.

One major challenge lies in the difficulty of accessing innovative treatments. Although (potentially) actionable genetic markers were detected for 81% of patients, 15% of them did not receive a treatment recommendation. Three primary reasons could be suggested. Firstly, drug accessibility after European Medicines Agency (EMA) approval might be delayed. A recent study on drug access in six European countries found that the time to access may differ in different countries of the E.U. and that hospitals in Belgium had slower access than Italy and France^26^. Secondly, clinical trials involving matched drugs may be conducted exclusively abroad, possibly not even within Europe. This underscores the importance of expert review by nMTB members actively involved in conducting clinical trials. An up-to-date clinical trial database for trials active in Belgium, as was recently established (cancertrials.be), will increase the usefulness of MTB recommendations. Of note, the BSMO PRECISION initiative might have itself played a role in attracting more clinical trials to Belgium^19^. Thirdly, in contrast to other countries where large clinical trials are being conducted to facilitate access to and evaluate the effectiveness of targeted and immunotherapy drugs, such as the DRUP trial in the Netherlands^27^, a comparable large-scale initiative is lacking in Belgium. In anticipation of establishing a similar pan-cancer multi-drug basket trial in Belgium, leveraging the insights and experience gained from BALLETT would be essential, as successful implementation of such an initiative would require widespread access to CGP.

Of the 69% of patients who received a CGP-based treatment recommendation, 23% received the matched treatment. A prominent reason for deviation was the physician’s choice, often due to prioritizing another treatment strategy. This might be attributed to the high frequency of variants with low potential clinical evidence (tier IID), often matched with less attractive Phase I/II clinical trials. Secondly, patients frequently rejected the recommendation, particularly if the proposed clinical trial was not available within an acceptable distance. This represents an additional challenge in accessing novel drugs and clinical trials and may underscore the need for a broader roll-out of clinical studies across Europe and across the country. In addition, our findings support the need for decentralized clinical trials, which would, however, require a major shift in the consideration, implementation, and conduct of cancer clinical trials^28^. Deteriorating health was another reason for deviation from nMTB recommendations, suggesting the need for NGS-informed treatment selection earlier in a patient’s disease course. Implementing CGP at diagnosis for all patients with advanced cancer could support better treatment strategies and early genome-directed therapies, rather than using them as a last resort. Window-of-opportunity treatments, given before exhausting standard options, could increase both the uptake and efficacy of these therapies.

Notably, the uptake of the treatment recommendations differed greatly among participating hospitals, with no uptake in one hospital. Reasons for this finding should be explored. Possibilities include lack of awareness and training of physicians and limited availability of an adequate clinical study infrastructure in the hospital, among other factors. These observations highlight the need for training in precision oncology and should drive national and local efforts toward improved clinical trial infrastructure and access.

Another major challenge encountered in this study involved standardizing treatment recommendations within the nMTB. The custom-designed app for providing clear data visualization to nMTB members emerged as indispensable, facilitating structured and harmonized discussions (Supplementary Fig. S10). Matching drugs and clinical trials to targets primarily involved manual searches of online databases and consultation with two different tertiary NGS data analysis systems that were available for the study. While these systems provide automated matching of targets to drugs and clinical trials, manual filtering and consideration of trial options are still required to ensure relevance and adequacy considering the patient’s cancer history. The development of a comprehensive, user-friendly, up-to-date, and effective decision support tool is crucial to aid healthcare professionals in interpreting complex genomic data and formulating evidence-based treatment recommendations. Such a tool would enhance the efficiency of the decision-making process and contribute to the harmonization of CGP-based interventions.

Using the findings of the present study to inform the Belgian healthcare system about reimbursement decisions also remains a challenge. In future work, we will explore whether patients have experienced benefits in terms of their PFS ratio upon the completion of patient follow-up. However, the likelihood of observing significantly improved clinical outcomes will be challenged by the fact that only a minority of patients received the matched treatment. Convincing stakeholders ideally also involves presenting data on compelling economic outcomes. It was previously reported that NGS testing led to annual cost savings of 25000 USD per patient due to reduced drug costs resulting from enrollment in clinical trials^29^. However, the cost-efficiency of CGP remains unclear and is a major barrier to reimbursement in many countries. In this study, the cost of CGP was estimated at 1.831,94 euros per test (incl. VAT). Going forward, the BALLETT study database will further be utilized for healthcare technology assessment in collaboration with the Netherlands Cancer Institute (NKI, Amsterdam) as part of the EU-funded Can.Heal project (Can.Heal - Building the EU genomics platform (canheal.edu)). These efforts aim to clarify the economic impact of CGP.

In conclusion, the BALLETT study not only reinforces the clinical utility of CGP in identifying actionable targets but also emphasizes the importance of collaborative efforts, standardized approaches, and comprehensive decision-making frameworks. It offers a potential framework for decentralized CGP implementation in other regions, though feasibility depends on local challenges and infrastructure. As precision oncology continues to evolve, our findings contribute to the growing body of literature advocating for integrating CGP into routine clinical practice. Addressing challenges related to innovative drug access, standardizing treatment recommendations with decision support tools, and evidence generation is imperative for the sustained integration of CGP into the Belgian healthcare system.

## Methods

### Study design and ethical approval

The PRECISION working group of BSMO designed the clinical study protocol which is available at clinicaltrials.gov (NCT05058937). The study was conducted under the precepts established in the Declaration of Helsinki, Good Clinical Practice guidelines, and all applicable regulatory requirements.

The study was approved by the local ethical committees of the 12 participating clinical sites (Supplementary Table S1) and confirmed after central review by the central ethical committee (Commission for Medical Ethics, University Hospital Ghent, EC study reference BC-08269). For registration of the study data, an electronic Case Report Form (eCRF) was developed on the Castor EDC platform (Amsterdam, The Netherlands).

### Clinical sites and patients

Patients were recruited from 12 Belgian hospitals, comprising 4 university hospitals and 8 general hospitals. All participating hospitals have a clinical trial infrastructure, though the level of activity and extent of capabilities may vary. Patients eligible for the project were adults (>18 y) with any solid tumor type, metastasized or locally advanced, either at diagnosis or at progression on a standard-of-care treatment. Early treatment lines were preferred to maximize the chance of therapeutic clinical trial enrollment. Patients were required to have a life expectancy of >12 weeks, an Eastern Cooperative Oncology Group (ECOG) Performance Status of ≤2, and no severe hematopoietic, renal, and/or hepatic dysfunction as assessed by the local principal investigator. Patients had to have a tissue biopsy available of less than two years old containing >10% tumor cells for CGP analysis. The number of enrolled breast, colorectal, and lung cancer cases was capped at 120 per tumor type to ensure a significant number of patients with less prevalent tumor types. An initial number of 864 slots were available. All patients gave written informed consent for the study. Patients were recruited from May 2021 to October 2023.

### CGP by the BALLETT NGS laboratory consortium

A consortium of nine Belgian NGS laboratories was installed. Every week, one CGP run of eight or 16 samples pooled from the 12 participating sites was performed, alternating in one of the nine NGS laboratories. These laboratories comply with the ISO15189 standard for medical laboratories as assessed by the Belgian Accreditation Instance and are recognized by the Belgian healthcare authorities to perform NGS for clinical oncology. The consortium had weekly meetings to discuss technical aspects and patient results and to promote standardization of testing and variant classification, which needs to be performed according to the Belgian ComPerMed guidelines (Commission Personalized Medicine)^30^.

For optimal standardization, the commercial, off-the-shelf TruSight Oncology 500 CGP kit (TSO500) (Illumina, Inc., San Diego, CA) was used in all laboratories. This kit was previously thoroughly validated for its diagnostic implementation by one of the BALLETT laboratories (Jessa Hospital, Hasselt). Methodology and validation results were described previously^31^. In short, TSO500 is a hybrid capture-based CGP assay that allows for the detection of SNVs and indels in 523 tumor-associated genes, CNVs of 59 genes, fusions with 55 cancer driver genes, as well as MSI and TMB. Starting with 40–80 ng DNA and 40–80 ng RNA extracted from formalin-fixed and paraffin-embedded (FFPE) samples revealed a precision and accuracy >99% for all variant types. The analytical sensitivity and specificity were at least 99% for SNVs, indels, CNVs, MSI, and fusions. For TMB, only values near the threshold of 10 mutations/Mb could result in divergent clinical interpretations (TMB-high versus TMB-low). The limit-of-detection for SNVs and indels was well below the set threshold of 5% variant allele frequency (VAF). The effectiveness of its adoption in a clinical diagnostic setting for CGP was further prospectively demonstrated in a consecutive series of 624 patients with cancer^31^. A micro-costing study estimated the total cost of the TSO500 CGP test, at €1832 (incl VAT). This covered sample preparation, DNA/RNA extraction, instruments, labor, all reagents and consumables, sequencing, bioinformatics, quality control, and validation.All other NGS labs implemented TSO500 after engaging in an Illumina proficiency test run that yielded satisfactory results across all nine labs. Five labs performed their analyses with the standard TSO500 protocol with pooling of eight samples (DNA + RNA) per run on a High Output v2.5 (300 cycles) flow cell and 2 × 101 bp paired-end sequencing on a NextSeq500/550Dx instrument (Illumina, Inc.). The other four labs used the TSO500 High-Throughput (TSO500-HT) protocol, pooling 16 samples (DNA + RNA) per run on an SP flow cell and 2 × 101 bp paired-end sequencing on a NovaSeq6000 instrument (Illumina, Inc.).

During the trial, HRD testing was added to the CGP analysis in four NGS labs and was performed in 100 patients enrolled after April 2023 in a tumor-agnostic way. The TSO500 HRD analysis assesses the relevant genomic scars including loss of heterozygosity (LOH), telomeric-allelic imbalance (TAI), and large-scale state transitions (LST), producing a genomic instability score (GIS) using a proprietary algorithm powered by Myriad Genetics. A GIS score of 42 or higher was considered HRD positive.

All sequencing data were analyzed using the Illumina TruSight Oncology 500 Local App software version 2.2. The sequencing data of the samples with HRD testing included were analyzed on an Illumina, Inc. on-premise DRAGEN server version 3 with the DRAGEN TruSight Oncology 500 Analysis Software version 2.1.

### National molecular tumor board

Within the BSMO PRECISION initiative framework, a virtual nMTB was set up for weekly patient discussions^19^. nMTB members are PRECISION working group members, investigators of the GeNeo and BALLETT studies, other interested expert oncologists, pathologists, clinical biologists, geneticists, molecular biologists, and bioinformaticians from all over Belgium. CGP-based treatment recommendations by the expert group could consist of (1) an approved treatment, (2) participation in a Phase I, II, or III clinical trial, (3) a drug in a medical need/compassionate use/early access program, or (4) off-label use of a drug. Likely incidental findings of a germline genetic aberration predisposing to cancer were identified and discussed in view of patient referral for germline genetic testing and genetic counseling. To allow optimal visualization of patient data and results during the nMTB, a custom interactive web dashboard was developed using the Shiny package in R. This application retrieves data directly from the eCRF, allowing for an efficient display and analysis of the information in a user-friendly interface. To facilitate standardization of target-drug and target-clinical trial matching during the nMTB, the laboratory investigators had access to the tertiary NGS data analysis tools OncoKDM (OncoDNA, Gosselies, Belgium) and Clinical Genomics Workspace (CGW; Velsera, Charlestown, MA).

### Workflow

**Fig. 5:**
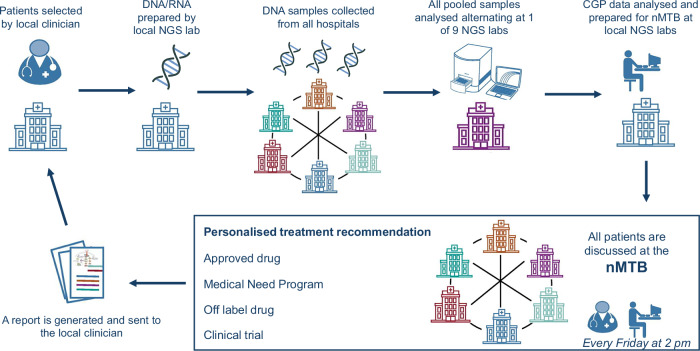
BALLETT study workflow. Workflow diagram, illustrating the process from patient recruitment to treatment recommendation.

The workflow is illustrated in Fig. 5. After patient recruitment by local oncologists, the local pathologist verified the availability of a suitable tissue biopsy. Subsequently, the local lab extracted DNA and RNA from FFPE slides using their accredited methods, and if the yield was sufficiently high the sample was registered into a custom-designed online sample registration and run scheduling (SRRS) tool. The SRRS tool then assigned the sample to a weekly CGP run, alternating across nine labs according to a predefined run schedule. All DNA/RNA samples were sent to the lab where the weekly CGP run was scheduled. After sequencing, the raw data were sent to the local labs from which the samples originated for further CGP data analysis.

All patient data, including demographics, tumor types, treatment history, and the CGP results, were registered in the eCRF by the local investigators and visualized for the nMTB members by the BALLETT Shiny app. The local investigators were invited to join the nMTB, often contributing additional clinical insights on their patients. After patient discussion in the nMTB, the treatment recommendations were also registered in the eCRF, and a report was automatically generated from the BALLETT app and sent to the local oncologist as advice for patient management, to be used at the treating physician’s discretion. The full content of this report is available in Supplementary Table S2. Enrollment in a therapeutic clinical trial requires the signature of the trial-specific informed consent form (ICF).

### Outcome measures

The outcome measures were of a descriptive nature:

1) Variants were clinically classified using the AMP/ASCO/CAP tiering system: Tier IA or IB, strong clinical significance, and Tier IIC and IID, potential clinical significance^32^. Tier IA, Tier IB, Tier IIC, and Tier IID variants were considered actionable, broadly aligning with the ESMO Scale for Clinical Actionability of Molecular Targets (ESCAT) tiers: I (ready for routine use), II (investigational), and III/IV (hypothetical)^33^.

The number and prevalence of actionable variants, categorized by type (SNVs/CNVs/fusions) and clinical tier using CGP were analyzed. This prevalence was compared to the prevalence of actionable variants if only the minimal tumor NGS panels required per tumor type in Belgium would have been used, which are restricted to genes with level 1 and level 2a designation according to ComPerMed.be (See Supplementary Table S3: evidence levels for diagnostic, prognostic or theragnostic biomarkers, as defined by ComPerMed.be).

2) Description of the patient journey, including the percentage of patients with successful CGP, the percentage of patients with MTB recommendation and by type of treatment recommendation, the percentage of patients accessing the CGP-based nMTB recommended treatment, and reason for deviating from the treatment recommendation (as assessed by the local teams at the end of the study), TAT from ICF signature to nMTB recommendation, and timing of treatment initiation following the nMTB recommendation.

## Supplementary information

Suppl Table S4

Supplementary Information

## Data Availability

The datasets generated and/or analyzed during the current study are available in the cBioPortal repository, https://www.cbioportal.org/study/summary?id=ballett_bsmo.

## Code availability

The code utilized for statistical analysis and figure creation in this study, written in R, is available upon request. Interested parties are encouraged to reach out for access, and we will provide the necessary files in a timely manner.
