# Supplementary material for: A nationwide comprehensive genomic profiling and molecular tumor board platform for patients with advanced cancer
Source: NPJ Precis Oncol. 2025 Mar 10;9:66. doi: 10.1038/s41698-025-00858-0 (PMC11893761; doi:10.1038/s41698-025-00858-0)
Supplement: Supplementary file 2 — Supplementary Information [file 41698_2025_858_MOESM2_ESM.docx]

**SUPPLEMENTARY INFORMATION**

**Unlocking precision oncology by establishing a nationwide comprehensive genomic profiling and molecular tumor board platform for patients with advanced cancer: the BALLETT study**

Pieter-Jan Volders, Philippe Aftimos, Franceska Dedeurwaerdere, Geert Martens, Jean-Luc Canon, Gabriela Beniuga, Guy Froyen, Jacques Van Huysse, Rebecca De Pauw, Hans Prenen, Suzan Lambin, Lore Decoster, Freya Vaeyens, Sylvie Rottey, Pieter-Jan Van Dam, Lynn Decoster, Annemie Rutten, Max Schreuer, Siebe Loontiens, Joni Van der Meulen, Jeroen Mebis, Kristof Cuppens, Sabine Tejpar, Isabelle Vanden Bempt, Jacques De Grève, David Schröder, Cédric van Marcke, Marc Van Den Bulcke, Evandro de Azambuja, Kevin Punie, Brigitte Maes

**Content**

[Figure S1: Distribution of histological subtypes across the tumor types included in the BALLETT study. 3](#_Toc190079506)

[Figure S2: CGP success rates across different tumor types in the BALLETT study. 4](#_Toc190079507)

[Figure S3: Frequency of genomic alterations in the top mutated genes across all tumor types in the BALLETT study. 5](#_Toc190079508)

[Figure S4: Distribution of the most-frequently observed genomic alterations by tumor type in the BALLETT study. 6](#_Toc190079509)

[Figure S5: Distribution of the number of genomic alterations per patient in the BALLETT study. 7](#_Toc190079510)

[Figure S6: Distribution of actionable markers across tiers with strong clinical significance (IA, IB), potential clinical significance (IIC and IID), and uncertain clinical significance (IIIA) by tumor type. 8](#_Toc190079511)

[Figure S7: Distribution of the number of different recommendations by the nMTB per patient. 9](#_Toc190079512)

[Figure S8: Reasons for deviating from treatment recommendations across participating hospitals in the BALLETT study. 10](#_Toc190079513)

[Figure S9: Distribution of cancer susceptibility gene (CSG) variants detected through CGP on tumor biopsies in the BALLETT study. 11](#_Toc190079514)

[Figure S10: Screenshot of the custom-designed BALLETT app for data visualization during national Molecular Tumor Board (nMTB) discussions. 12](#_Toc190079515)

[Table S1: Local approving ethics committees of all participating sites. 13](#_Toc190079516)

[Table S2: Template of nMTB report outlining the full content. 14](#_Toc190079517)

[Table S3: Evidence levels for diagnostic, prognostic or theragnostic biomarkers, according to the Belgian healthcare system (ComPerMed). 16](#_Toc190079518)

[Table S4: Full list of actionable biomarkers resulting in a treatment recommendation. 17](#_Toc190079519)

[Table S5: Actionable biomarkers that did not lead to a treatment recommendation. 18](#_Toc190079520)

# Figure S1: Distribution of histological subtypes across the tumor types included in the BALLETT study.

**
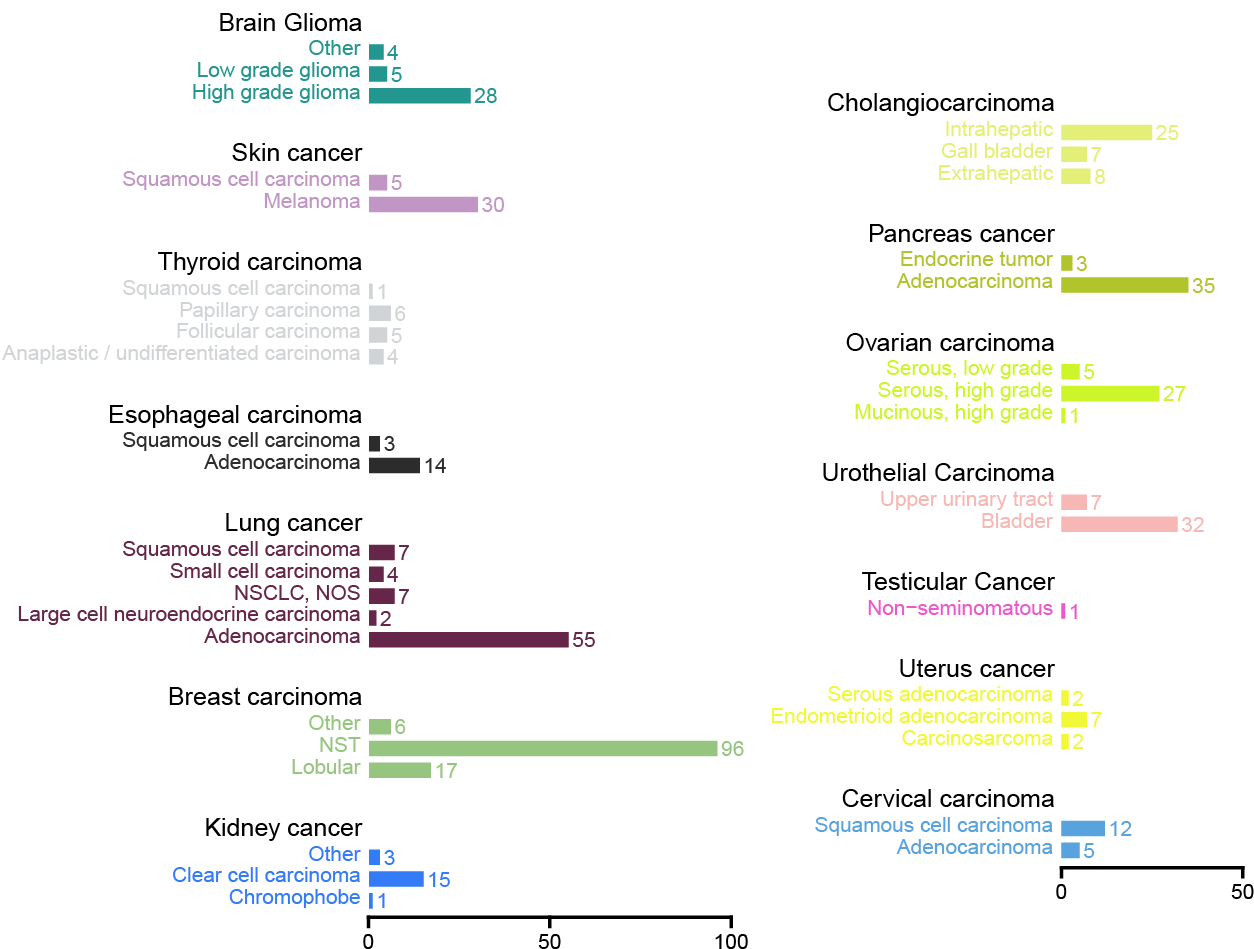
**

# Figure S2: CGP success rates across different tumor types in the BALLETT study.

The success rate ranges from 71% for uveal melanoma to 100% for thymoma and neuroendocrine tumor with an overall success rate of 89%.


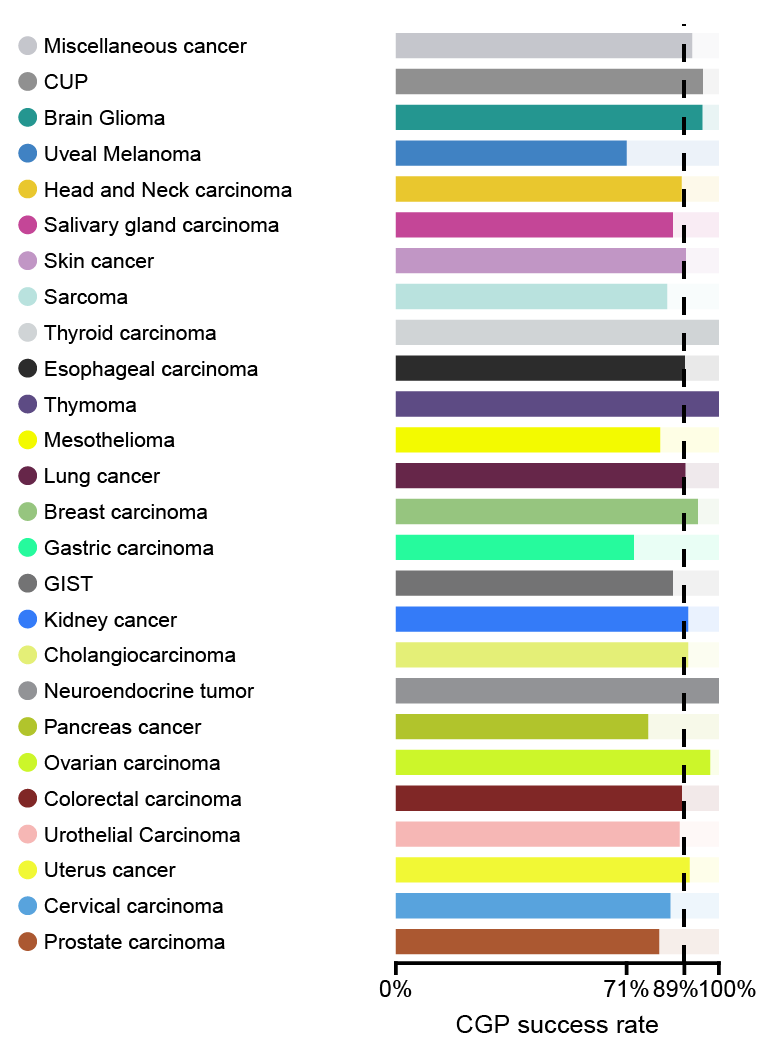


# Figure S3: Frequency of genomic alterations in the top mutated genes across all tumor types in the BALLETT study.


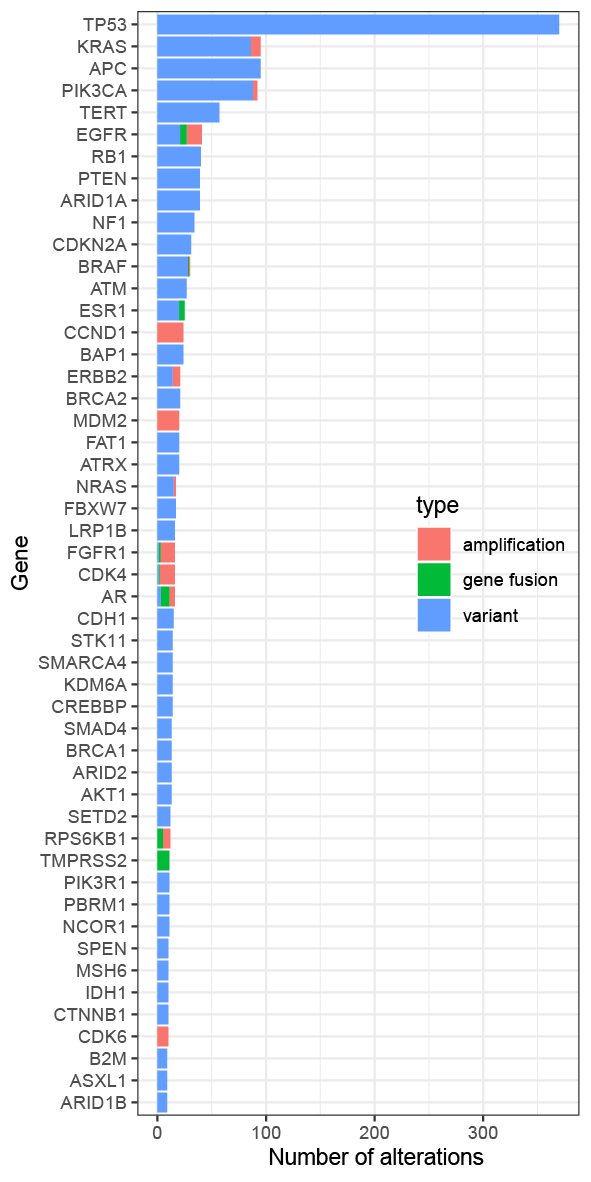


# Figure S4: Distribution of the most-frequently observed genomic alterations by tumor type in the BALLETT study.


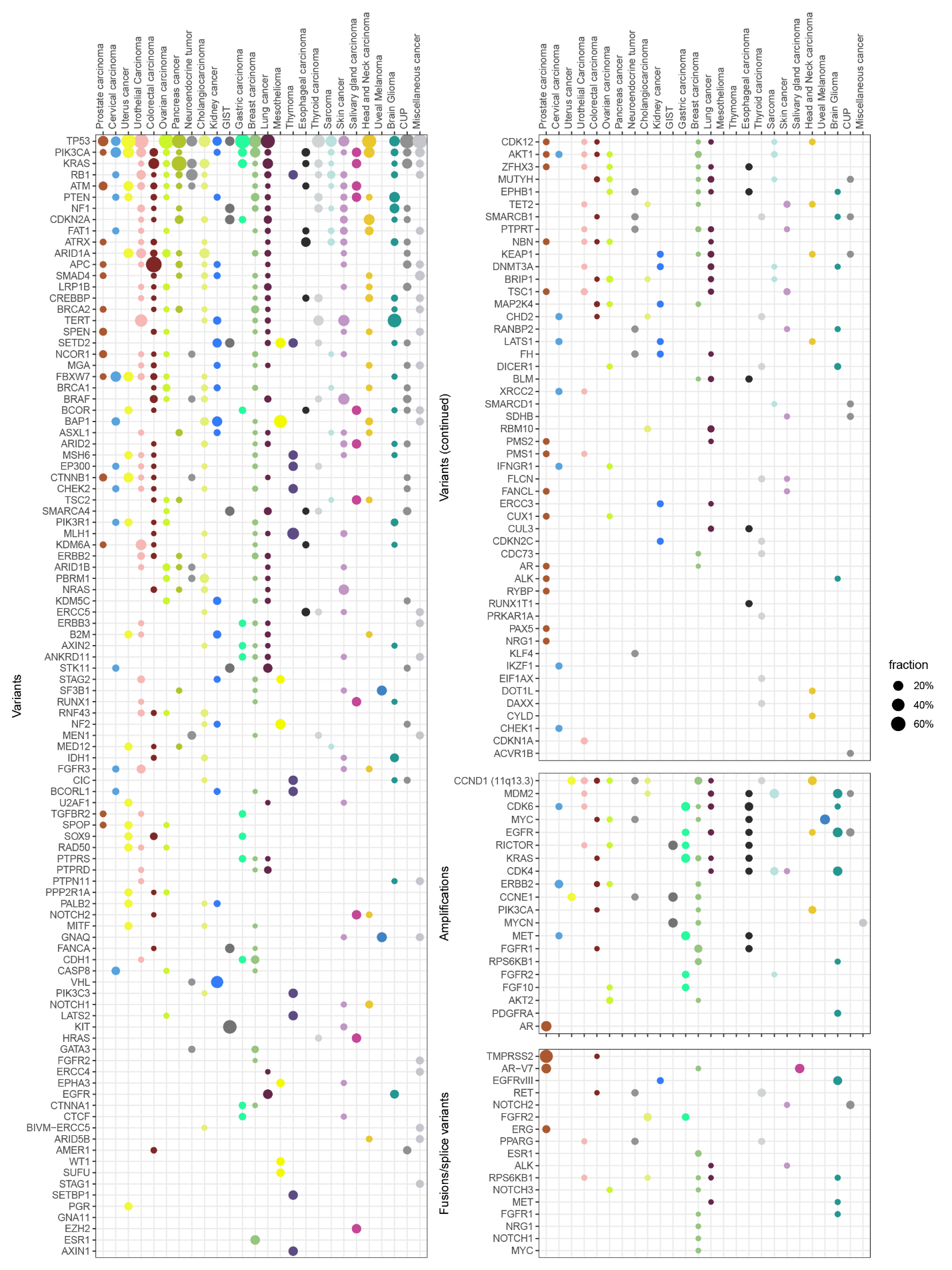


# Figure S5: Distribution of the number of genomic alterations per patient in the BALLETT study.


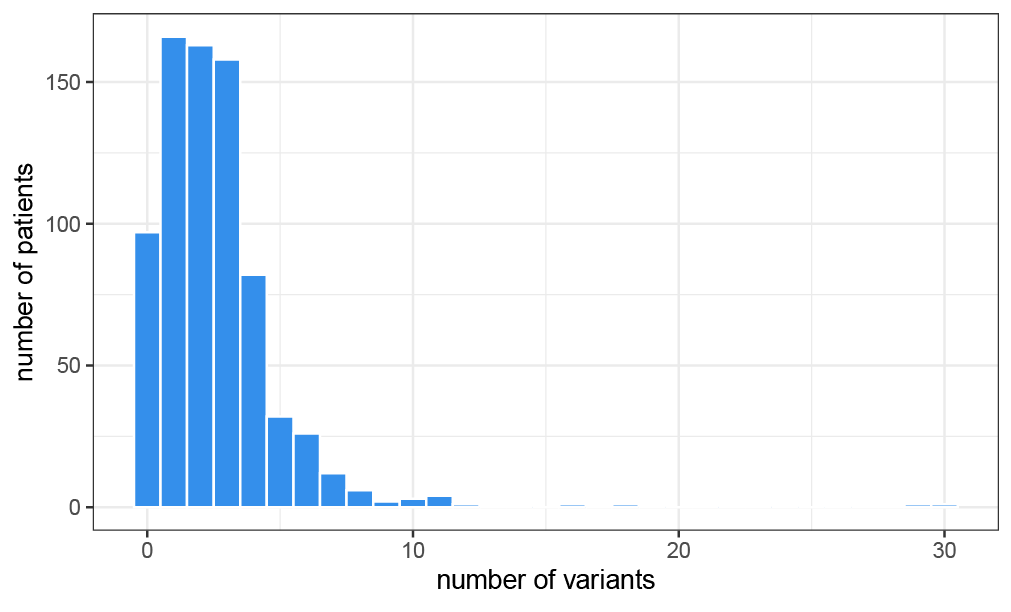


# Figure S6: Distribution of actionable markers across tiers with strong clinical significance (IA, IB), potential clinical significance (IIC and IID), and uncertain clinical significance (IIIA) by tumor type.

**
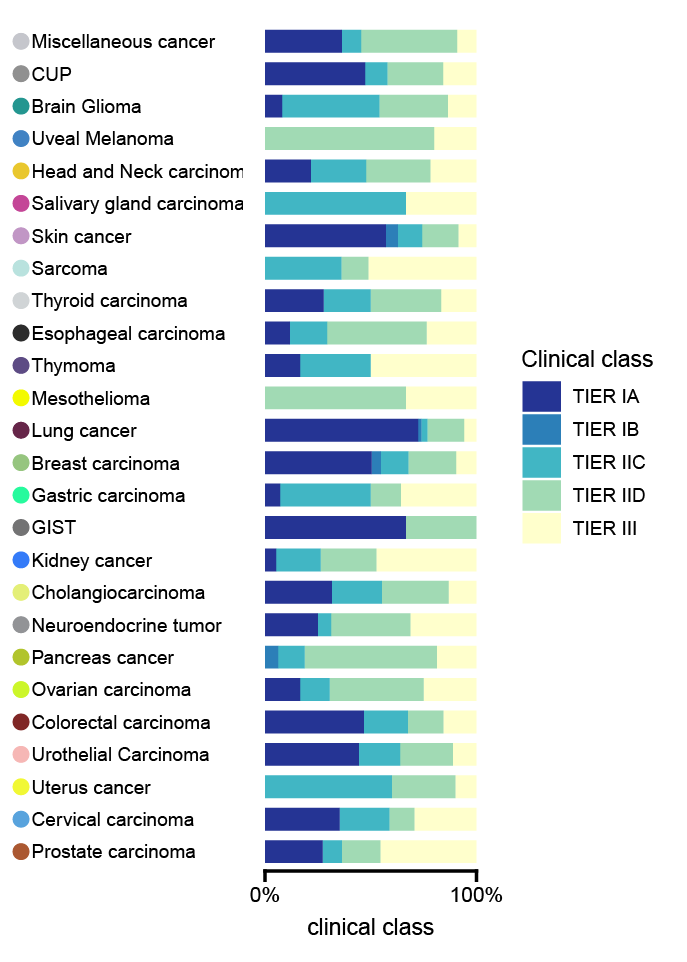
**

# **Figure S7: Distribution of the number of different recommendations by the nMTB per patient**.


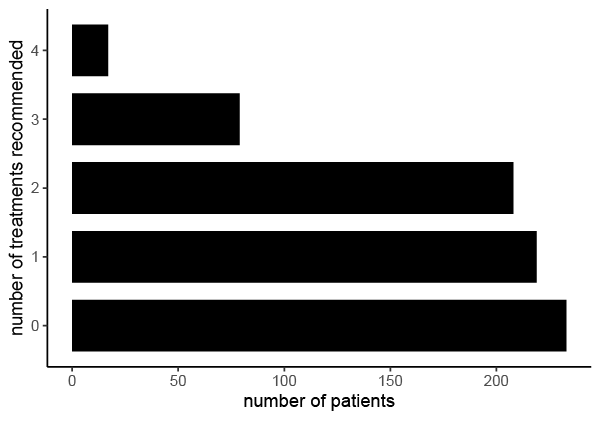


# Figure S8: Reasons for deviating from treatment recommendations across participating hospitals in the BALLETT study.


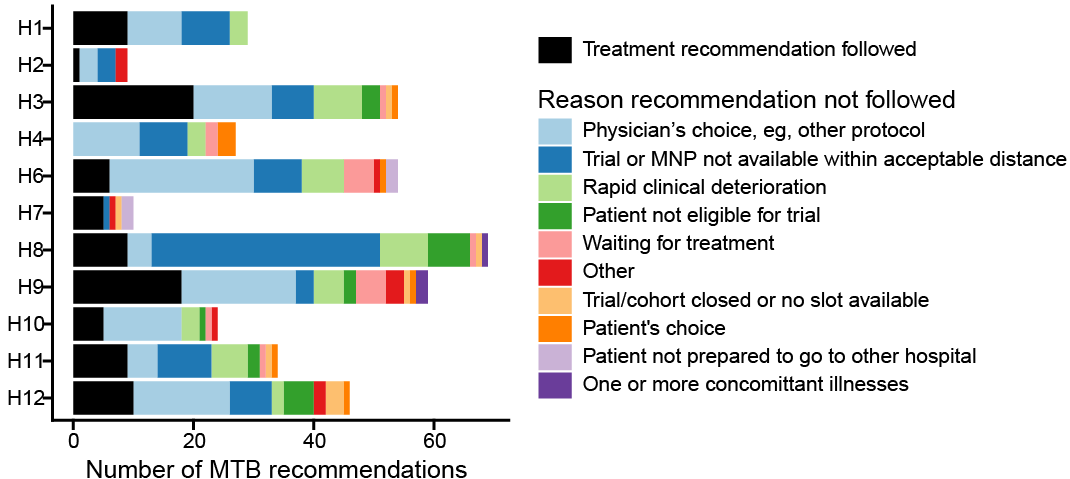


# Figure S9: Distribution of cancer susceptibility gene (CSG) variants detected through CGP on tumor biopsies in the BALLETT study.

Only genes from the ESMO Precision Medicine Working Group 2023 recommendations are shown (Kuzbari et al).


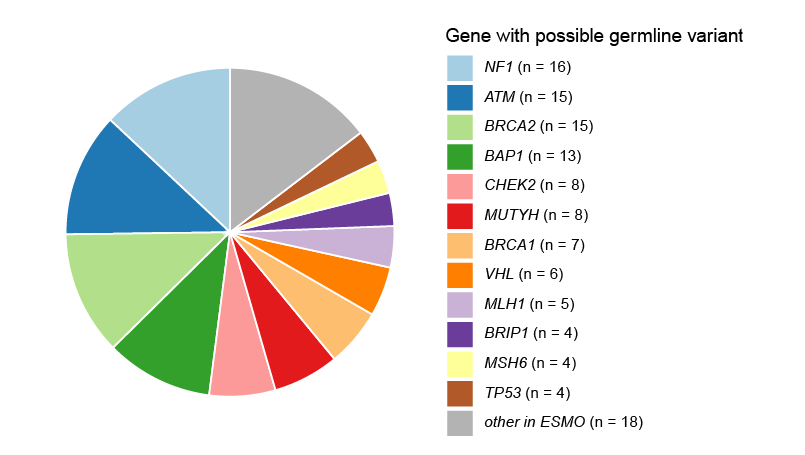


# Figure S10: Screenshot of the custom-designed BALLETT app for data visualization during national Molecular Tumor Board (nMTB) discussions.


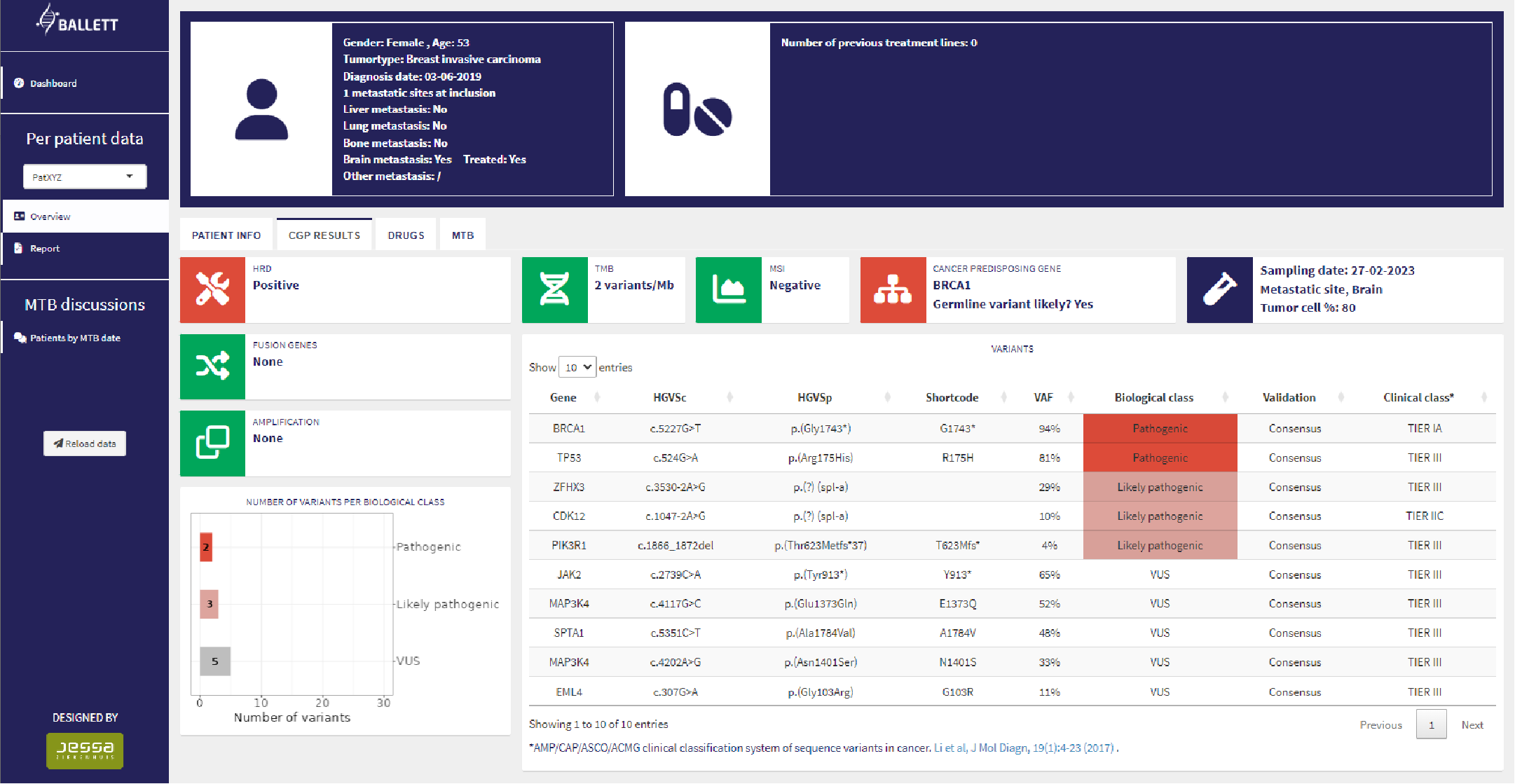


# Table S1: Local approving ethics committees of all participating sites.

| **Participating site** | **Ethics committee** |
| --- | --- |
| UZ Gent | Commissie voor Medische Ethiek |
| UZ Brussel | Commissie Medische Ethiek |
| UZA | Ethisch Comité |
| ZNA | Commissie voor Medische Ethiek ZNA |
| AZ Turnhout | Medisch Ethisch Comité |
| GZA | Ethische commissie GZA |
| Grand Hôpital de Charleroi | Comité d'Ethique |
| Jessa Ziekenhuis | Ethische toetsingscommissie |
| UZ Leuven | Ethische Commissie Onderzoek UZ Leuven |
| AZ Sint-Jan Brugge | Commissie voor ethiek |
| AZ Delta Roeselare | Commissie medische ethiek |
| ASZ Ziekenhuis Aalst | Commissie voor Medische Ethiek |

# Table S2: Template of nMTB report outlining the full content.

Date: …

To Dr. …

BALLETT patient ID …..

Dear colleague,

Your patient was discussed by the BALLETT laboratory working group on (date) and during the Molecular Tumor Board on (date).

Please find below the summary of the patient data, the results of the ‘Comprehensive Genomic Profiling’ (performed by the TSO500 kit of Illumina) as well as the therapy recommendation(s) based on the genomic results. Please consider these recommendation(s) in view of all detailed patient factors, treatment history, contraindications, other therapy options and patient’s preferences. The final treatment decision (including clinical trial eligibility assessment) is the full and sole responsibility of the treating physician.

**Patient summary**

Gender: …..

Age: ….

Tumor type: ….

Diagnosis date: ….

Metastasis: Yes/No

(number) metastatic sites at inclusion

Liver metastasis: Yes/No

Lung metastasis: Yes/No

Bone metastasis: Yes/No

Brain metastasis: Yes/No

Other metastasis: ….

**CGP results**

***Sample summary***

Sampling date: …

(Primary) or (Metastatic site)

Tumor cell percentage: ….

***Analysis results***

**TMB**

…. variants/Mb

**MSI**

….

**HRD**

….

**CANCER PREDISPOSING GENES:**

….

Germline variant likely? Yes/No

**FUSION GENES: …….**

**AMPLIFICATIONS: ……**

**VARIANTS:**

| **Gene** | **HGVSc** | **HGVSp** | **Shortcode** | **VAF** | **Biological class** | **Validation** | **Clinical class*** |
| --- | --- | --- | --- | --- | --- | --- | --- |
|  |  |  |  |  |  |  |  |
|  |  |  |  |  |  |  |  |

* AMP/CAP/ASCO/ACMG clinical classification system of sequence variants in cancer. Li et al, J Mol Diagn, 19(1):4-23 (2017).

**Markers for matched therapy**

***Target – drug match***

| **Gene** | **Pathway** | **Drug class** | **Drug name** |
| --- | --- | --- | --- |
|  |  |  |  |
|  |  |  |  |

***Immunotherapy drugs***

***……***

**Therapy recommendations**

***Priority 1***

…….

***Priority 2***

……

***Priority 3***

…….

**Disclaimers:**

• This recommendation is based on the molecular profile of the tumor and does not account for other patient factors. The final treatment decision is the full and sole responsibility of the treating physician.

• The incidental finding of a germline variant may occur, but the analysis does not allow to discriminate between somatic and germline variants. Additional germline testing is required for confirmation or exclusion of germline variants.

Sincerely,

**The BALLETT Molecular Tumor Board Members**

For questions or additional information:

Local NGS laboratory or Dr. Brigitte Maes, coordinating head investigator of BALLETT: Brigitte.Maes@jessazh.be, tel: 011 33 83 41

# Table S3: Evidence levels for diagnostic, prognostic or theragnostic biomarkers, according to the Belgian healthcare system (ComPerMed).

| Level | Description |
| --- | --- |
| 1 | - Standard of care biomarker for diagnosis and/or prognosis^1^ - Biomarker predictive of a response or resistance to a reimbursed drug in Belgium for this indication |
| 2A | - Recommended standard of care biomarker for diagnosis and/or prognosis^2^ - Biomarker predictive of response or resistance to an EMA-approved drug for this indication |
| 2B | - Biomarker predictive of response or resistance to an EMA-approved drug for another indication or to a drug for which a clinical trial is available in this indication |
| 3 | - Preliminary clinical evidence supporting the biomarker for diagnosis and/or prognosis^3^ - Biomarker predictive of a response or resistance to a drug for which a clinical trial is not available in this indication or to a compassionate use of drug |

1 Standard of care: Included in guidelines (e.g., WHO, ELN, NCCN) and consensus from ComPerMed experts

2 Recommended standard of care: Clinical evidence and consensus from experts ComPerMed

3 Preliminary evidence and no consensus from ComPerMed experts

# Table S4: Full list of actionable biomarkers resulting in a treatment recommendation.

See separate Excel File

# Table S5: Actionable biomarkers that did not lead to a treatment recommendation.

| **Cancer type** | **Gene** | **Marker** | **Clinical class** | **Number of cases** |
| --- | --- | --- | --- | --- |
| Breast carcinoma | *CCND1* (11q13.3) | amplification | TIER IID | 7 |
| Breast carcinoma | *MYC* | amplification | TIER IID | 5 |
| Breast carcinoma | *ESR1* | variant | TIER IA | 3 |
| Breast carcinoma | *PIK3CA* | variant | TIER IB | 3 |
| Colorectal carcinoma | *MYC* | amplification | TIER IID | 3 |
| Head and Neck carcinoma | *CDKN2A* | variant | TIER IID | 3 |
| Urothelial Carcinoma | *KDM6A* | variant | TIER IID | 3 |
| Brain Glioma | *EGFR* | amplification | TIER IID | 2 |
| Brain Glioma | *IDH1* | variant | TIER IIC | 2 |
| Breast carcinoma | *BRCA2* | variant | TIER IIC | 2 |
| Breast carcinoma | *FGFR1* | amplification | TIER IIC | 2 |
| Breast carcinoma | *PIK3CA* | variant | TIER IA | 2 |
| Cholangiocarcinoma | *KRAS* | variant | TIER IID | 2 |
| Colorectal carcinoma | *FGFR1* | amplification | TIER IIC | 2 |
| Colorectal carcinoma | *NRAS* | variant | TIER IA | 2 |
| Head and Neck carcinoma | *CCND1* (11q13.3) | amplification | TIER IID | 2 |
| Head and Neck carcinoma | *MYC* | amplification | TIER IID | 2 |
| Head and Neck carcinoma | *PIK3CA* | variant | TIER IIC | 2 |
| Lung cancer | *EGFR* | amplification | TIER IID | 2 |
| Ovarian carcinoma | *CCND1* (11q13.3) | amplification | TIER IID | 2 |
| Ovarian carcinoma | *CCNE1* | amplification | TIER IID | 2 |
| Ovarian carcinoma | *MYC* | amplification | TIER IID | 2 |
| Urothelial Carcinoma | *ATM* | variant | TIER IIC | 2 |
| Brain Glioma | *CDK4* | amplification | TIER IID | 1 |
| Brain Glioma | *EGFR* | variant | TIER IID | 1 |
| Brain Glioma | *KDM6A* | variant | TIER IID | 1 |
| Brain Glioma | *PIK3CA* | variant | TIER IIC | 1 |
| Brain Glioma | *PTEN* | variant | TIER IIC | 1 |
| Breast carcinoma | *ARID1A* | variant | TIER IID | 1 |
| Breast carcinoma | *BRCA1* | variant | TIER IA | 1 |
| Breast carcinoma | *CCNE1* | amplification | TIER IID | 1 |
| Breast carcinoma | *CDH1* | variant | TIER IID | 1 |
| Breast carcinoma | *ERBB2* | amplification | TIER IA | 1 |
| Breast carcinoma | *ESR1* | amplification | TIER IIC | 1 |
| Breast carcinoma | *ESR1* | gene fusions | TIER IID | 1 |
| Breast carcinoma | *FGFR2* | variant | TIER IID | 1 |
| Breast carcinoma | *TP53* | variant | TIER IID | 1 |
| Cervical carcinoma | *FBXW7* | variant | TIER IID | 1 |
| Cervical carcinoma | *PIK3CA* | variant | TIER IIC | 1 |

| *Table S5 continued* | |  |  |  |  |
| --- | --- | --- | --- | --- | --- |
| **Cancer type** | | **Gene** | **Marker** | **Clinical class** | **Number of cases** |
| Cholangiocarcinoma | *ARID1A* | variant | TIER IIC | 1 |  |
| Cholangiocarcinoma | *FBXW7* | variant | TIER IID | 1 |  |
| Colorectal carcinoma | *ATM* | variant | TIER IIC | 1 |  |
| Colorectal carcinoma | *CCND1* (11q13.3) | amplification | TIER IID | 1 |  |
| Colorectal carcinoma | *CDKN2A* | variant | TIER IID | 1 |  |
| Colorectal carcinoma | *EGFR* | amplification | TIER IID | 1 |  |
| Esophageal carcinoma | *CCND1* (11q13.3) | amplification | TIER IID | 1 |  |
| Esophageal carcinoma | *EGFR* | amplification | TIER IID | 1 |  |
| Esophageal carcinoma | *FGFR1* | amplification | TIER IIC | 1 |  |
| Esophageal carcinoma | *KRAS* | variant | TIER IID | 1 |  |
| Esophageal carcinoma | *MYC* | amplification | TIER IID | 1 |  |
| Esophageal carcinoma | *SMARCA4* | variant | TIER IID | 1 |  |
| Gastric carcinoma | *CCND1* (11q13.3) | amplification | TIER IID | 1 |  |
| Gastric carcinoma | *MYC* | amplification | TIER IID | 1 |  |
| Head and Neck carcinoma | *IDH2* | variant | TIER IIC | 1 |  |
| Kidney cancer | *VHL* | variant | TIER IID | 1 |  |
| Lung cancer | *CCNE1* | amplification | TIER IID | 1 |  |
| Lung cancer | *EGFR* | variant | TIER IA | 1 |  |
| Lung cancer | *KIT* | amplification | TIER IID | 1 |  |
| Lung cancer | *PDGFRA* | amplification | TIER IID | 1 |  |
| Lung cancer | *PIK3CA* | variant | TIER IIC | 1 |  |
| Mesothelioma | *NF2* | variant | TIER IID | 1 |  |
| Miscellaneous cancer | *CCND1* (11q13.3) | amplification | TIER IID | 1 |  |
| Ovarian carcinoma | *BRCA1* | variant | TIER IA | 1 |  |
| Pancreas cancer | *CCND1* (11q13.3) | amplification | TIER IID | 1 |  |
| Pancreas cancer | *KRAS* | variant | TIER IID | 1 |  |
| Pancreas cancer | *SRC* | variant | TIER IID | 1 |  |
| Penile cancer | *CCND1* (11q13.3) | amplification | TIER IID | 1 |  |
| Prostate carcinoma | *AR* | amplification | TIER IID | 1 |  |
| Prostate carcinoma | *FGFR1* | amplification | TIER IIC | 1 |  |
| Prostate carcinoma | *MYC* | amplification | TIER IID | 1 |  |
| Prostate carcinoma | *PMS2* | variant | TIER IID | 1 |  |
| Salivary gland carcinoma | *HRAS* | variant | TIER IIC | 1 |  |
| Salivary gland carcinoma | *NOTCH2* | variant | TIER IID | 1 |  |
| Salivary gland carcinoma | *PIK3CA* | variant | TIER IIC | 1 |  |
| Sarcoma | *CDK4* | amplification | TIER IID | 1 |  |
| Sarcoma | *FGFR1* | amplification | TIER IIC | 1 |  |
| Sarcoma | *FGFR3* | amplification | TIER IIC | 1 |  |
| Sarcoma | *MDM2* | amplification | TIER IIC | 1 |  |
| Sarcoma | *PDGFRA* | amplification | TIER IID | 1 |  |
| *Table S5 continued* |  |  |  |  |  |
| **Cancer type** | **Gene** | **Marker** | **Clinical class** | **Number of cases** |  |
| Sarcoma | *TP53* | variant | TIER IID | 1 |  |
| Sarcoma | *TSC2* | variant | TIER IIC | 1 |  |
| Skin cancer | *CCND1* (11q13.3) | amplification | TIER IID | 1 |  |
| Skin cancer | *CDKN2A* | variant | TIER IID | 1 |  |
| Skin cancer | *EGFR* | amplification | TIER IID | 1 |  |
| Thyroid carcinoma | *CCND1* (11q13.3) | amplification | TIER IID | 1 |  |
| Thyroid carcinoma | *CREBBP* | variant | TIER IID | 1 |  |
| Thyroid carcinoma | *EGFR* | amplification | TIER IID | 1 |  |
| Thyroid carcinoma | *HRAS* | variant | TIER IIC | 1 |  |
| Thyroid carcinoma | *KRAS* | variant | TIER IID | 1 |  |
| Urothelial Carcinoma | *ARID1A* | variant | TIER IID | 1 |  |
| Urothelial Carcinoma | *CCND1* (11q13.3) | amplification | TIER IID | 1 |  |
| Urothelial Carcinoma | *MYC* | amplification | TIER IID | 1 |  |
| Urothelial Carcinoma | *NF1* | variant | TIER IID | 1 |  |
| Urothelial Carcinoma | *PIK3CA* | variant | TIER IIC | 1 |  |
| Uterus cancer | *MYC* | amplification | TIER IID | 1 |  |
| Vulva Carcinoma | *CDKN2A* | variant | TIER IID | 1 |  |
| Vulva Carcinoma | *PIK3CA* | variant | TIER IIC | 1 |  |
